# An Empirical Comparison of Human Value Models

**DOI:** 10.3389/fpsyg.2018.01643

**Published:** 2018-09-25

**Authors:** Paul H. P. Hanel, Lukas F. Litzellachner, Gregory R. Maio

**Affiliations:** Department of Psychology, University of Bath, Bath, United Kingdom

**Keywords:** human values, altruistic behavior, general health behavior, pro-environmental behavior, cultural values

## Abstract

Over the past century, various value models have been proposed. To determine which value model best predicts prosocial behavior, mental health, and pro-environmental behavior, we subjected seven value models to a hierarchical regression analysis. A sample of University students (*N* = 271) completed the Portrait Value Questionnaire (Schwartz et al., 2012), the Basic Value Survey (Gouveia et al., 2008), and the Social Value Orientation scale (Van Lange et al., 1997). Additionally, they completed the Values Survey Module (Hofstede and Minkov, 2013), Inglehart’s (1977) materialism–postmaterialism items, the Study of Values, fourth edition (Allport et al., 1960; Kopelman et al., 2003), and the Rokeach (1973) Value Survey. However, because the reliability of the latter measures was low, only the PVQ-RR, the BVS, and the SVO where entered into our analysis. Our results provide empirical evidence that the PVQ-RR is the strongest predictor of all three outcome variables, explaining variance above and beyond the other two instruments in almost all cases. The BVS significantly predicted prosocial and pro-environmental behavior, while the SVO only explained variance in pro-environmental behavior.

## Introduction

Over the past century, many models of human values have been proposed and empirically supported. While all developers of new value models have explained the theoretical advantages of their approach, they have not tested whether their value model has a higher predictive validity than any of the previous value models. In the present research, we aim to address this gap by directly comparing whether more recent value models are better in explaining a range of behaviors than their predecessors and whether they predict behaviors differently. We first give a very brief history of models of values, before turning to our selection of value models for comparison and summarizing the scarce literature comparing value models (Rokeach, 1973; Spates, 1983; Rohan, 2000; Hitlin and Piliavin, 2004; Gouveia, 2013; Maio, 2016).

### History of Human Values

Human values are often defined as abstract ideals that guide people’s behavior (Schwartz, 1992; Maio, 2010; Fischer, 2017). Here, we describe value models that have been cited frequently in the literature and build at least partly on each other: Spranger’s (1921) model of types of people as operationalized by Vernon and Allport (1931) and Allport et al. (1960), Rokeach’s (1973) instrumental and terminal values, Schwartz’s (Schwartz, 1992; Schwartz et al., 2012) quasi-circumplex-model of human values, and Gouveia’s (2013) functional theory of values. Further, we included two prominent cultural value models: Inglehart’s (1977) materialism–postmaterialism values and Hofstede’s (1980, 2001) cultural value dimensions. Additionally, we measured social value orientations (SVOs; Van Lange et al., 1997). We first describe models that measure values on an individual basis, before turning to cultural value models.

### Individual Value Models

**FIGURE 1:**
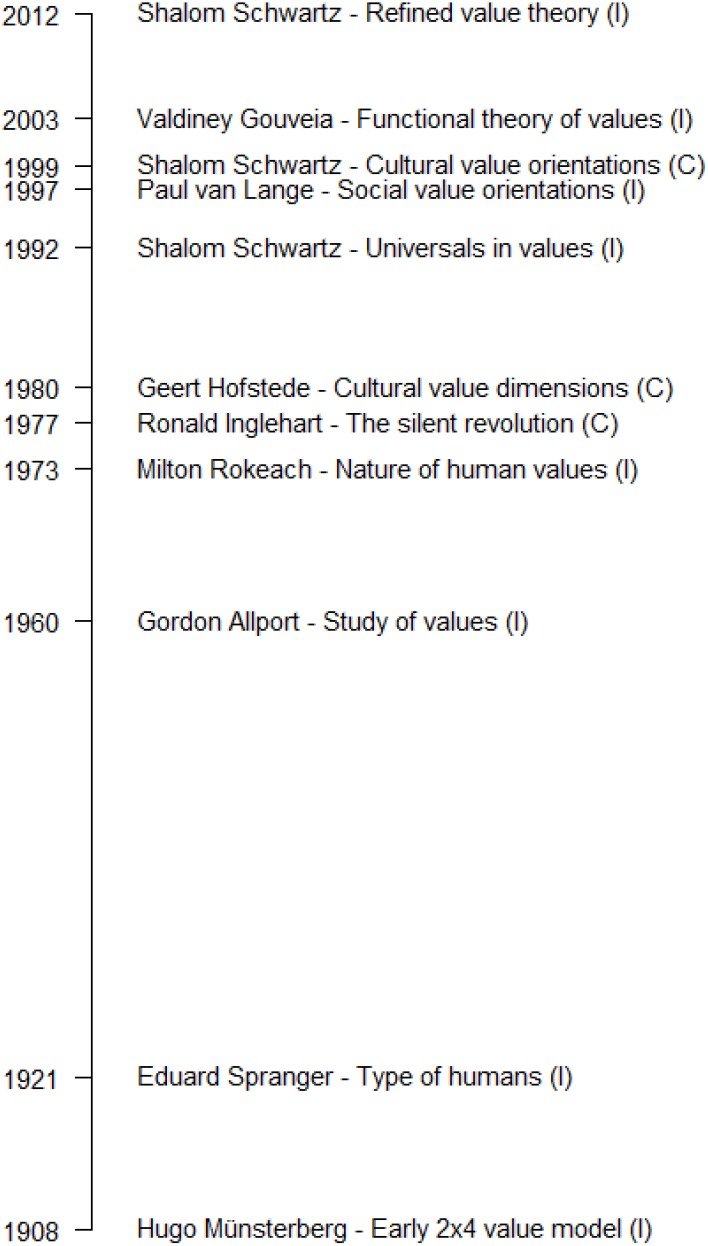
Historical overview of selected important contributions to human value research.

Early in the 20th century, Münsterberg (1908) provided the first formal psychological model of values. His model maps values onto a four-by-two framework. This framework contrasts life values with cultural values on one dimension and logical, aesthetic, ethical, and metaphysical values, on the other dimension. In each of the eight cells, there is one ‘value type’ and three values, which are further differentiated according to whether they relate to the external, social, and internal world. The value types are existence, unity, developmental, and god values (life value types), and coherence, beauty, achievement, and basic values (cultural value types). Münsterberg’s model appears to be somewhat similar to Gouveia’s functional theory, which we describe below (Gouveia, 2013; Gouveia et al., 2014a), although the names of the value types and values differ. However, to the best of our knowledge, there is no measure of values based on Münsterberg’s model (see **Figure 1** for a timeline of the here discussed value models).

Another important theoretical contribution was made by Eduard Spranger. In his book, *Types of Men*, Spranger (1921) differentiated between the theoretical, economic, aesthetic, social, political, and religious person. Spranger proposed that one of six value orientations is predominant in each person. The theoretical person, for example, strives for knowledge and truth, whereas the economic person strives for usefulness. Spranger influenced Vernon and Allport (1931) and Allport et al. (1960), who created a survey to measure the six types of values. In this survey, participants would first indicate their preference on a number of controversial statements before ranking other statements related to the six types.

However, Rokeach (1973) criticized Allport et al.’s (1960) measure of values as measuring attitudes, rather than idealized standards with an “ought” character (Maio, 2010). Instead, Rokeach proposed to measure values with 36 items such as “Equality (brotherhood, equal opportunity for all)” or “Obedient (dutiful, respectful),” which should be ranked based on their importance as guiding principles in one’s life. Using this approach, Rokeach obtained abundant evidence for links between the importance of values and diverse attitudes and behavior (e.g., Rokeach, 1971, 1973).

Rokeach’s (1973) model was highly influential, but it lacked a method for making predictions about connections between *different* values and other attitudes and behavior. Schwartz and Bilsky (1987, 1990) built on Rokeach’s conceptualization of values, but introduced a theoretical perspective examining motivational differences between values. These researchers found that Rokeach’s 36 values could be ordered into seven or eight value types based on their motivational dynamics and that these value types, in turn, can be organized into a two-dimensional circumplex. Building on these findings and a new theoretical perspective, Schwartz (1992) postulated the existence of 11 value types and assessed these in the Schwartz Value Survey. The 11 value types are self-direction, stimulation, hedonism, achievement, power, security, tradition, conformity, spirituality, benevolence, and universalism. Based on analyses conducted by Roccas and Sagiv, Schwartz (1992) found across 20 countries and 40 samples (mostly students and teachers) that 10 value types could be reliably differentiated in most samples. Each value type consists of two to nine values and can be ordered along two dimensions: openness versus conservation and self-transcendence versus self-enhancement. Spirituality did not emerge as an independent value type. During the last 25 years, in samples from more than 80 countries, Schwartz et al. (2001, 2012) and Bilsky et al. (2011) have found support for his proposed structure of human values.

Schwartz et al. (2012) published a revised version of his theory, postulating 19 rather than 10 value types. Most of the 10 value types were divided into two. For example, the value type self-direction was divided into self-direction-thoughts and self-direction-actions (see **Table 1** for the 19 value types and conceptual definitions). In his refined theory, Schwartz introduces a contrast between values with a personal focus (openness and self-enhancement) and those with a social focus (conservation and self-transcendence), thereby incorporating a contrast proposed 60 years earlier (Parsons et al., 1951). It is worth noting that this model’s new sub-divisions of the motivational value types (Gouveia et al., 2014a) can be justified by arguing that the values form a motivational continuum. Just as the color spectrum can be divided into very few or very many categories, so too can the array of values (Schwartz, 2014). Schwartz’s theory has been validated with a range of measures (Schwartz, 1992; Schwartz et al., 2001, 2012; Lee et al., 2008; for an overview, see Roccas et al., 2017) and methods (Schwartz, 1992; Schwartz and Boehnke, 2004; Coelho et al., 2018). Further, picture-based value surveys for children found that the proposed structure replicated even among children as young as 5-years (Döring et al., 2010; Collins et al., 2017; Lee et al., 2017).

**Table 1:** Values and definitions in Schwartz’s revised model of values.

Value	Conceptual definitions in terms of motivational goals
Self-Direction–Thought	Freedom to cultivate one’s own ideas and abilities
Self-Direction–Action	Freedom to determine one’s own actions
Stimulation	Excitement, novelty, and change
Hedonism	Pleasure and sensuous gratification
Achievement	Success according to social standards
Power–Dominance	Power through exercising control over people
Power–Resources	Power through control of material and social resources
Face	Security and power through maintaining one’s public image and avoiding humiliation
Security–Personal	Safety in one’s immediate environment
Security–Societal	Safety and stability in the wider society
Tradition	Maintaining and preserving cultural, family, or religious traditions
Conformity–Rules	Compliance with rules, laws, and formal obligations
Conformity–Interpersonal	Avoidance of upsetting or harming other people
Humility	Recognizing one’s insignificance in the larger scheme of things
Benevolence–Dependability	Being a reliable and trustworthy member of the ingroup
Benevolence–Caring	Devotion to the welfare of ingroup members
Universalism–Concern	Commitment to equality, justice, and protection for all people
Universalism–Nature	Preservation of the natural environment
Universalism–Tolerance	Acceptance and understanding of those who are different from oneself

*Adapted from Schwartz et al. (2012).*

The most recent value theory is Gouveia’s functional theory of human values (Gouveia, 2013; Gouveia et al., 2014a), which has been developed and tested during the last 15 years. It builds on Maslow’s (1943) theory and is based on two functions of values, whether values express needs (survival vs. thriving needs) or guide actions (personal vs. central vs. social goals), mapped in a two-by-three framework (**Table 2**). The functional theory was challenged by Schwartz (2014) as not being distinct from his own value theory (Schwartz, 1992). Indeed, several findings that are based on the functional theory (e.g., Fischer et al., 2011; Gouveia et al., 2015) could also have been obtained using Schwartz’s theory. Nevertheless, the functional theory includes fewer value types, while covering dimensions similar to those in Schwartz’s model (Gouveia et al., 2014a,b). The structure of the functional theory was also replicated among children (Gouveia et al., 2011).

**Table 2:** The functional theory of human values.

		Values as guides of actions (circle of goals)		
		Personal goals	Central goals	Social goals
Values as expressions of needs (level of needs)	*Thriving needs*	**Excitement values**	**Suprapersonal values**	**Interactive values**


		Emotion	Beauty	Affection
		Pleasure	Knowledge	Belonging
		Sexuality	Maturity	Support
	*Survival needs*	**Promotion values**	**Existence values**	**Normative values**
		Power	Health	Obedience
		Prestige	Stability	Religiosity
		Success	Survival	Tradition

*Adapted from Gouveia et al. (2014a).*

Most of the above described value models build theoretically on each other, while a separate line of research has proceeded independently. This line of research investigated SVOs, which are “defined as stable preferences for certain patterns of outcomes for oneself and others” (Van Lange et al., 1997). Building on earlier research on motivations in mixed motive games (McClintock and Liebrand, 1988), Van Lange et al. (1997) distinguished between competitive, individualistic, and prosocial value orientations, which are usually measured by a point-allocation task in which participants need to allocate points to themselves and another person. Competitive individuals seek to maximize their own outcomes relative to the outcomes of the other person. Individualistic individuals seek to maximize their own outcomes, irrespective of the outcomes for the other person. Finally, prosocial individuals seek to strive for equality, while trying to maximize the outcomes for themselves *and* the other person. There are correlations between these classifications and scores on Schwartz’s measures of values (Joireman and Duell, 2005), but no comparison of the predictive power of these models to our knowledge.

### Cultural Value Models

The value theories described so far have focused on values at the individual level. However, as Kluckhohn (1951) noted, values can also be described on a cultural level. Three prominent approaches of this type were proposed by Inglehart (1977), Hofstede (1980), Hofstede (2001), and Schwartz (1999, 2006). Inglehart reduced Maslow’s (1943) hierarchy of needs to materialism and postmaterialism, which are at opposite ends of a unidimensional continuum (for discussions about the unidimensionality, see Saccbi, 1998; Dobewall and Rudnev, 2014). Materialism is linked with physical and material security, whereas postmaterialism is linked with freedom and self-expression. Inglehart was especially interested in whether and how values shift across time in relation to cultural change. For example, he found that economic growth is accompanied by a shift in values from materialism to post-materialism (Inglehart and Baker, 2000). In other words, societies become more tolerant, rational, and focus less on absolute norms when they economically develop. Importantly, Inglehart’s measure of materialism and postmaterialism values can also be assessed on an individual level to test for individual differences, rather than country or cultural differences.

Hofstede links values and culture in his work on cultural dimensions. His understanding of values is based on the research of Kluckhohn (1951) and Rokeach (1973). His definition of culture, “the collective programming of the mind that distinguishes the members of one group or category of people from another” (Hofstede, 2001), points directly to the aim of his empirical work: to find elements that reliably differentiate cultures. Based on work he conducted for a computing corporation, IBM, Hofstede (1980) identified four cultural dimensions, which he later extended to six dimensions: Power–distance, individualism vs. collectivism, uncertainty avoidance, masculinity vs. femininity, long-term orientation, and indulgence vs. restraints (Hofstede, 2001; Hofstede et al., 2010). Importantly, Hofstede argued that the cultural dimensions can only be found on a country but not on an individual level (cf. Fischer et al., 2010). However, others found that Hofstede’s values can be measured on an individual level and developed a suitable scale for this (Yoo et al., 2011).

Hofstede’s perspective has been highly influential in cross-cultural research, but Schwartz’s (2006) theory of cultural value orientations (CVOs) has recently emerged as a useful alternate view. Schwartz’s CVO model is derived from his prior theory of individual human values (Schwartz, 1992), which was described above. In other words, the CVOs consists of the same values as the original individual-level model containing 10 value types (Schwartz, 1992), but grouped together in a way that better reflect cultural idiosyncrasies. Schwartz (2006) posits seven *a priori* CVOs, which are thought to “express shared conceptions of what is good and desirable in the culture, the cultural ideals” (Schwartz, 2006). In his approach, cultural values are usually measured by aggregating value scores from individual responses to measures of values in a culture. The seven CVOs are intellectual autonomy (being independent), affective autonomy (pursuing positive affective experiences), mastery (encouraging self-assertion), hierarchy (unequal distribution of power), embeddedness (being part of a collective), harmony (fitting harmoniously into the environment), and egalitarianism (being concerned for others).

### Comparisons of Value Models

The above described value models show a range of similarities and differences (for more extensive comparisons of at least two value models, see Schwartz, 2006; Datler et al., 2013). To help compare the models in brief, it is useful to use Schwartz’s model as reference point because of its high contemporary influence. One of the most salient differences between Schwartz’s refined value model (Schwartz et al., 2012) and the other value models is the number of proposed values. For example, whereas Inglehart’s (1977) value model consists of only two values, Schwartz’s refined theory (Schwartz et al., 2012) consists of 19 value types. The materialism–postmaterialism dimension of Inglehart is highly correlated with Schwartz’s embeddedness-autonomy (or security/tradition- self-direction) and Hofstede’s collectivism–individualism dimension (Inglehart and Oyserman, 2004; Dobewall and Strack, 2014). However, the materialism–postmaterialism dimension of Inglehart misses out on a range of other important values that can be found in other value models, such as benevolence or power values (Dobewall and Rudnev, 2014) or interactive and promotion values (in Gouveia’s model). Similarly, the SVOs (Van Lange et al., 1997) do not capture values related to security or tradition. The values of Gouveia’s (2003) model do, as discussed above, tap into motivational dimensions very similar to those expressed by Schwartz’s values (Gouveia et al., 2014b; Schwartz, 2014), although they do not capture explicitly self-direction values. For example, Gouveia’s excitement values are very similar to Schwartz’s stimulation and hedonism values, promotion is very similar to achievement and power, existence is very similar to security, and normative to tradition and conformity (see Lima, 2012, for empirical support). Lima (2012) found slightly stronger correlations between behavior intentions and self-reported behaviors with the values of the functional theory than with Schwartz’s values. Also, Rokeach values are very similar to Schwartz’s (1992) values, as Schwartz used the 36 values proposed by Rokeach as a basis of his model. However, Rokeach’s values do not reflect an *a priori* theoretical distinction between values expressing different motives, and there is no empirical basis for confirming its comprehensiveness. (It can be argued that the capture of the circular space by Schwartz’s model shows comprehensiveness, at least for two value dimensions.) In contrast, Spranger’s (1921) types of values are more distinct. While the economic, political, religious, and social values overlap partly with achievement, power, tradition, and benevolence values, the aesthetic and theoretical values are more conceptually distinct from Schwartz’s values (which do not explicitly separate artistic and intellectual orientations).

Although these conceptual comparisons align with past descriptions of the value models, empirical data comparing the models is sparse. Surprisingly, only a few studies have compared value models directly, and researchers have rarely compared more than two value models. A direct comparison between Schwartz’s (1992) original 10 value type model and Inglehart’s model (Inglehart and Baker, 2000) revealed that Inglehart’s value measure had lower internal consistency and construct validity, but it was more consistent in predicting a range of dependent variables such as openness to immigration, church attendance, or life satisfaction than Schwartz’s value measure (Datler et al., 2013). On a cultural level, Schwartz’s CVOs were related to international trade, whereas Hofstede’s cultural dimensions were not (Ng et al., 2007). In contrast, Schwartz’s CVOs were related to various dimensions of peacefulness approximately as strongly as were Hofstede’s and Inglehart’s values (Basabe and Valencia, 2007). There is a lack of comparisons across a range of models in the same studies. This omission is significant, particularly given the importance of values in research on diverse topics (e.g., environment, health, prejudice, social conflict). It would be useful to *empirically* determine how the models compare. In particular, do any of the models fare better in predicting important behaviors? Do some of the values described in other models fall into space neglected by Schwartz’s value model, which is arguably the most comprehensive thus far?

### The Present Research

In the present research, we tested which out of the seven previously described value models would be best in predicting three important behaviors on an individual level. Also, we tested whether more recent models explain variance above and beyond older models. We chose the values operationalized by Vernon and Allport (1931) and Allport et al. (1960), Rokeach (1973), Gouveia (2013), and Schwartz’s refined value model (Schwartz et al., 2012), because each of these models explicitly builds on earlier models. Additionally, we added Inglehart’s (1977) materialism–postmaterialism values and Hofstede’s (1984, 2001) cultural dimensions because of their prominence in cross-cultural research, including marketing research (e.g., Wong, 2004), political sciences and sociology (Inglehart and Baker, 2000). As a seventh value measure, we included the Social Value Orientations scale (Van Lange et al., 1997). Although it is somewhat distinct from the other six value measures (both in conceptualizations and measurement), we included the measure to test whether the SVOs explain variance in prosocial behavior, one of the three selected dependent variables, above and beyond the other six value measures (see rationale below).

We acknowledge that there have been several other studies that have compared other value measures, which we have not included in our survey. Examples are the List of Values and various work value measures (Beatty et al., 1985; Ravlin and Meglino, 1987; Oishi et al., 1998). They were not included because our survey was already long. We were worried that adding additional scales would have decreased the reliabilities of the measures because of fatigue.

As dependent variables, we selected prosocial, environmental, and mental health behaviors because of their relevance in contemporary research. Also, all of those behaviors were found to be related to values in past research (Kristiansen, 1985; Bond and Chi, 1997; Schultz and Zelezny, 1998; de Miranda Coelho et al., 2006; Sagiv et al., 2011; Bilican et al., 2016; Hanel and Wolfradt, 2016; Bouman et al., 2018). Based on this previous research and theoretical considerations, we predicted that prosocial value orientations (Van Lange et al., 1997) would be the strongest predictor of prosocial behavior, because the point allocation task used to measure SVOs is a kind of prosocial behavior in itself. Furthermore, we assumed that identifying oneself with the social value type (Spranger, 1921; Allport et al., 1960), and placing higher importance on benevolence values (Schwartz et al., 2012) and interactive values (Gouveia, 2013) are also associated with prosocial behavior. In addition, although we postulated that Schwartz et al.’s (2012) universalism–nature value type would be the strongest predictor of environmental behavior because of its underlying motivation to preserve the natural environment, we expected that long-term orientation (Hofstede et al., 2010) would also be related to pro-environmental behavior because environmental pollution has mostly long-term consequences. Finally, we predicted that security-personal values (Schwartz et al., 2012) and existence values (Gouveia, 2013) are the strongest predictors of health related behavior, because both reflect the importance that individuals place on personal safety and health.

In a final step, we tested whether we could replicate Lima’s (2012) findings that there are meaningful correlations between Gouveia’s and Schwartz’s values (see above) and subjected all value types into a single multidimensional space analysis.

## Materials and Methods

### Respondents

Our sample consisted of 271 psychology undergraduate students from Cardiff University who responded to an online survey in exchange for course credits. For the hierarchical regressions, 35 respondents were excluded due to missing data (it is not possible to have different sample sizes in hierarchical regression analysis across variables). Of these participants, 22 were excluded due to having an ambiguous SVO (they did not choose any specific option more than six times), the other 13 due to missing data in the outcome variables leaving 236 (212 women; *M*_age_ = 20.35 years, *SD* = 9.38) respondents within the hierarchical regression analyses. The study was approved by the ethics committee of the School of Psychology, Cardiff University.

### Procedure and Measurements

The survey was constructed and presented online. After receiving information about the survey’s aims and giving consent, respondents were asked to answer different scales assessing their values. Additionally, the survey contained instruments measuring prosocial behavior, mental health, and environmental behavior. The questionnaires and their items were presented in a randomized order. Respondents were debriefed upon completion of the survey.

### Value Instruments

To measure the 19 values of the revised Schwartz model, we used the revised Portrait Value Questionnaire (PVQ-RR, Schwartz et al., 2012). This instrument asks respondents to read 57 statements about a fictitious person of the same gender as them. The statements convey information about what the other person considers to be important in life, such as “It is important to her to care for nature” (universalism–nature). For each individual statement, respondents are asked to indicate how similar they are to that other person. The response scale ranged from 1 (not at all like me) to 6 (very much like me). The items were averaged to represent 19 value types (Schwartz et al., 2012), which had reliabilities between α = 0.59 (humility) and α = 0.87 (universalism–nature).

The values of the functional theory were measured with the Basic Value Survey (Gouveia, 2003; Gouveia et al., 2008). Respondents rated the importance of each of 18 values (e.g., social support, religiosity, belonging) using a scale ranging from 1 (completely unimportant) to 7 (of the utmost importance). The six resulting value types were adequately reliable, with αs ranging from 0.56 (suprapersonal) to 0.69 (interactive).

Social value orientations were assessed using the 9-item measure (Van Lange et al., 1997). The measures asked participants to choose among three options in which they simultaneously assign points to themselves and to another, unknown person. Every item presented an option to either maximize their own outcome (individual value orientation), maximize the point difference between themselves and the other (competitive), or split the points evenly between both players (prosocial). Choosing one of these options at least six out of nine times assigns a respondent to an individualistic (*N* = 41), a competitive (*N* = 7), or a prosocial value orientation (*N* = 177), respectively. In line with previous literature (Joireman et al., 2004; Joireman and Duell, 2005), we grouped seven respondents with a competitive SVO together with the individualists to form a pro-self group. To be able to compare pro-selfs to prosocials in subsequent analyses, we utilized dummy coding.

Other value measurements included in the survey where the Values Survey Module (Hofstede and Minkov, 2013), the Rokeach (1973) Value Survey, the Studies of Values (Kopelman et al., 2003) which is an updated version of Allport et al.’s (1960) measure of Spranger’s (1921) value types, and the Materialism–Postmaterialism Scale (Inglehart and Flanagan, 1987). However, due to low reliabilities (Values Survey Module: maximum α = 0.29; SOV: maximum α = 0.26; Materialism Scale: average correlation between the two parts: *r* = 0.36), or multicollinearity issues (RVS), we dismissed them from all further analyses. Thus, we only focused on the PVQ-RR, the BVS, and the SVO-task in the analysis.

### Behavior Scales

Prosocial behavior was assessed with the self-reported Altruism Scale (Rushton et al., 1981). Respondents read 20 statements about past pro-social behavior (e.g., “I have given money to a charity,” “I have given a stranger a lift in my car”). To indicate how frequently they carried out these acts, respondents used a 5-point Likert-scale ranging from “Never” to “Very often.” Their score was then created by averaging all items (α = 0.84).

Respondents’ health was assessed by the 12 question short-form of the General Health Questionnaire (Goldberg and Williams, 1988). It asks the respondents about their mental well-being over the last few weeks (e.g., “have you recently lost much sleep over worry?”, “have you recently felt you couldn’t overcome your difficulties?”). These questions were answered on a 4-point Likert-scale. The answer options were always phrased so that high scores indicate low mental well-being (α = 0.89). Because the content of the questions revolves around mental rather than general health, we will henceforth refer to the construct measured by this scale as *mental health*.

Pro-environmental behavior was measured with 12 items that were used in previous studies (Whitmarsh and O’Neill, 2010; Evans et al., 2014). Participants indicated how often they behaved in an environmentally friendly manner (e.g., “turned off lights you’re not using,” “Walk, cycle or used public transportation for short journeys of less than 5 km”). Responses were given on a 5-point scale ranging from “Never” to “Very often.” Alternatively, respondents could indicate that this activity does not apply to them. The overall score was computed by summing across all items and dividing by the number of items that applied to the participant (α = 0.69).

### Statistical Analysis

We used the R software environment (R Development Core Team, 2017) for statistical computing. The analysis script can be found alongside the data^1^. We conducted 18 hierarchical regressions (six per outcome) to investigate our hypotheses regarding each behavior. We determined whether a newly added value measure significantly predicts variance left unexplained by the previously included value measures by comparing the amount of variance explained, adjusted for the number of predictors within the analysis (adjusted *R*^2^), before and after a measure was added to the analysis. We used the adjusted *R*^2^ to account for the measures each consisting of an unequal number of predictors. To investigate the relations among the value measures themselves, we subjected the BVS and the PVQ-RR to a Multidimensional Scaling (MDS) Analysis.

## Results

**Table 3** shows the means and standard deviations for all outcome measures per SVO. Contrary to previous research (Joireman et al., 2004), *t*-tests revealed a significant differences in pro-environmental behavior between respondents with a prosocial SVO (*M* = 3.35) and respondents with an pro-self SVO (*M* = 3.09), *t*(61.58) = 2.27, *p* = 0.027, *r* = 0.21. Other differences between the SVO groups were negligible.

**Table 3:** Means and standard deviations of all outcome variables for both social value orientations.

Value orientation	Pro-social behavior	Mental health	Environmentalism
Prosocial (*N* = 189)	2.70 (0.50)	1.31 (0.49)	3.35 (0.59)
Pro-Self (*N* = 47)	2.68 (0.62)	1.30 (0.50)	3.09 (0.73)

*Numbers in brackets represent the standard deviation.*

**Table 4** shows the correlations among all value types from the three instruments remaining the analysis. As in previous literature (e.g., Lima, 2012), we observed strong associations between normative BVS values and the PVQ-RR values of conformity and tradition, with tradition showing an exceptionally large correlation of *r* = 0.71. Furthermore, the BVS value existence was most strongly related to the PVQ-RR value security, and the BVS value excitement was associated with the PVQ-RR values stimulation and hedonism. The BVS value promotion and the PVQ-RR values of Power and Achievement were also highly correlated. Less straight forward was the relationship between the BVS value interactive and the PVQ-RR values. The BVS interactive value was highly correlated with security and most self-transcendent values. Finally, suprapersonal BVS values were mostly related to PVQ-RR values that represented openness (such as self-direction – action) or self-transcendence (such as universalism – concern).

**Table 4:** Correlations of all value measures within the analysis.

PVQ-RR	BVS						SVO Prosocial
	Interactive	Normative	Suprapersonal	Existence	Excitement	Promotion	
Self-Direction–Thought	0.12	-0.15	0.32	0.29	0.26	0.17	0.07
Self-Direction–Action	0.16	-0.02	0.33	0.27	0.29	0.23	0.10
Stimulation	0.14	0.04	0.30	0.20	0.47	0.25	0.04
Hedonism	0.38	-0.05	0.25	0.29	0.56	0.19	-0.06
Achievement	0.44	0.11	0.37	0.38	0.37	0.54	-0.17
Power–Dominance	0.02	0.21	0.08	0.09	0.04	0.59	-0.10
Power–Resources	0.02	0.13	0.07	0.06	0.15	0.54	-0.17
Face	0.28	0.23	0.25	0.23	0.10	0.37	-0.14
Security–Personal	0.41	0.30	0.16	0.47	0.08	0.24	-0.14
Security–Societal	0.31	0.29	0.21	0.29	0.26	0.23	-0.07
Tradition	0.10	0.71	0.03	0.15	-0.01	0.20	-0.03
Conformity–Rule	0.22	0.47	0.05	0.26	-0.12	0.14	-0.04
Conformity–Interpersonal	0.29	0.33	0.17	0.22	-0.02	0.05	-0.03
Humility	0.28	0.25	0.17	0.25	0.05	0.02	0.14
Universalism–Nature	0.06	0.05	0.29	0.14	0.13	0.04	0.10
Universalism–Concern	0.31	0.04	0.36	0.24	0.13	0.00	0.24
Universalism–Tolerance	0.30	0.11	0.30	0.27	0.10	0.01	0.21
Benevolence–Care	0.42	0.20	0.15	0.33	0.12	0.04	0.06
Benevolence–Dependence	0.37	0.20	0.24	0.32	0.21	0.26	-0.02
SVO–Prosocial	-0.10	-0.07	-0.02	-0.08	-0.09	-0.17	1

*Correlations with the SVO are biserial correlations. Positive scores indicate a positive relationship with a prosocial SVO. All correlations >0.13 are significant at p < 0.05, correlations >0.17 at p < 0.01, correlations >0.22 at p < 0.001.*

Interestingly, only the BVS value promotion was negatively related to the SVO, while several self-enhancement or self-transcendence PVQ-RR values were significantly associated with the SVO.

**Table 5** shows the correlations between the value types and the outcome variables. Both value instruments showed small-to-medium size correlations with pro-social behavior. For the PVQ-RR, the highest correlation was found for universalism–nature, *r* = 0.29; for the BVS, the highest correlation was for suprapersonal values, *r* = 0.23. There were only a few small correlations with mental health: the strongest correlations for the PVQ-RR occurred for face, *r* = 0.27, conformity, *r* = 0.15 and hedonism, *r* = 0.16; for the BVS, only significant correlation was with suprapersonal values, *r* = 0.16. There were small-to-large correlations with pro-environmental behavior. The highest correlations for the PVQ-RR were with universalism–nature, *r* = 0.48, and the highest correlations for the BVS were with suprapersonal values, *r* = 0.26. In any case, the correlations of the PVQ-RR for all three outcome variables tended to be larger than those of the BVS.

**Table 5:** Correlations of all PVQ-RR and BVS values with all outcome variables.

Value	Pro-social behavior	Mental health	Environmentalism
**PVQ**			
Self-direction–Thought	0.12	-0.07	0.15
Self-direction–Action	0.17	-0.07	0.16
Stimulation	0.13	-0.01	0.13
Hedonism	-0.02	-0.16	0.01
Achievement	0.08	-0.02	0.00
Power–Dominance	0.16	0.06	0.03
Power–Resources	0.01	0.04	-0.03
Face	0.09	0.27	0.10
Security–Personal	-0.01	-0.09	0.01
Security–Societal	0.15	0.00	0.08
Tradition	0.23	-0.03	0.05
Conformity–Rule	0.11	-0.01	0.08
Conformity–Interpersonal	0.05	0.15	0.16
Humility	0.21	-0.06	0.15
Universalism–Nature	0.29	0.09	0.48
Universalism–Concern	0.20	0.08	0.22
Universalism–Tolerance	0.21	0.06	0.22
Benevolence–Care	0.17	-0.04	0.11
Benevolence–Dependence	0.20	0.00	0.07
SVO–Prosocial	0.02	0.01	0.17
**BVS**			
Interactive	0.06	-0.07	0.01
Normative	0.17	-0.04	0.06
Suprapersonal	0.23	0.16	0.26
Existence	0.13	-0.08	0.07
Excitement	0.07	-0.05	-0.01
Promotion	0.15	-0.00	0.02

*Correlations with the SVO are biserial correlations. Positive scores indicate a positive relationship with a prosocial SVO. All correlations >0.13 are significant at p < 0.05, correlations >0.17 at p < 0.01, correlations >0.22 at p < 0.001.*

To test which value measure explains the biggest portion of variance in each individual outcome variable, we conducted 18 regressions (six per outcome). For every outcome variable, we entered all the values from each measure in the first step of three separate regressions (e.g., all PVQ-RR values in one step), followed by entry in the second step of the remaining two measures in one order (e.g., the BVS and then SVO). We then repeated the three regressions using the alternate order of the remaining measures in the second step (e.g., the SVO and then the BVS).

### Hierarchical Regressions – Prosocial Behavior

When entered first, the PVQ-RR and the BVS explained 14% (adjusted *R*^2^ = 0.14, *p* < 0.001) and 6% (adjusted *R*^2^ = 0.06, *p* = 0.001) of the observed variance in prosocial behavior, respectively. The SVO did not explain a significant amount of variance on its own (adjusted *R*^2^< 0.01, *p* = 0.75).

Entering any other values on top of the PVQ-RR did not significantly increase the proportion of variance explained (BVS: adjusted *R*^2^ = 0.15, *p* = 0.27 [amount of explained variance by BVS + PVQ-RR; *p*-value refers to *R*^2^ change]; SVO: adjusted *R*^2^ = 0.14, *p* = 0.37). Entering the BVS in the first step and the PVQ-RR in the second step lead to a significant increase in explained variance (adjusted *R*^2^ = 0.15, *p* = 0.005). Similarly, entering the PVQ in the second step explained variance beyond the SVO (adjusted *R*^2^ = 0.14, *p* < 0.001). Entering the BVS in the second step after the SVO increased the amount of variance explained by 6% (adjusted *R*^2^ = 0.06, *p* < 0.001).

The PVQ-RR still explained additional variance after both BVS and SVO were entered (adjusted *R^2^* = 0.15, *p* = 0.004). Entering the SVO after both PVQ-RR and BVS did not led to a significant increase in explained variance (adjusted *R*^2^ = 0.15, *p* = 0.41), and neither did entering the BVS in the last step (adjusted *R*^2^ = 0.15, *p* = 0.28). Overall, the 19 basic values of the PVQ-RR were the best predictors of prosocial behavior.

### Hierarchical Regressions – Mental Health

The PVQ-RR was a significant predictor of variance in mental health (adjusted *R*^2^ = 0.16, *p* < 0.001). While SVO did not explain a significant amount of variance on its own (adjusted *R*^2^< 0.01, *p* = 0.91), the BVS missed the conventional level of significance only by a small margin (adjusted *R*^2^ = 0.03, *p* = 0.055). As neither of the other two measures where reliable predictors of mental health, we focus on the PVQ-RR for this part of the analysis.

Entering the PVQ-RR on top of the BVS still resulted in an increase of roughly 14% in explained variance (adjusted *R*^2^ = 0.17, *p* < 0.001). The same holds when entering it after the SVO (adjusted *R*^2^ = 0.16, *p* < 0.001), where it explains an additional 13% of variance. When entered last, the PVQ-RR still explains additional 14% variance on top of both BVS and SVO (adjusted *R*^2^ = 0.17, *p* < 0.001). No other value instruments significantly explained more variance when entered in the last step (BVS: adjusted *R*^2^ = 0.17, *p* = 0.19; SVO: Adjusted *R*^2^ = 0.17, *p* = 0.72). Overall, the Schwartz values were the best predictors of mental health.

### Hierarchical Regressions – Environmentalism

Respondents’ environmental behavior was significantly explained by all three value instruments (PVQ-RR: adjusted *R*^2^ = 0.23, *p* < 0.001; BVS: adjusted *R*^2^ = 0.08, *p* < 0.001; SVO: adjusted *R*^2^ = 0.02, *p* = 0.011). Entering the PVQ-RR in the second step increased the amount of explained variance by 16% when entering it on top of the BVS (adjusted *R*^2^ = 0.24, *p* < 0.001) as well as 22% on top of the SVO (adjusted *R*^2^ = 0.24, *p* < 0.001). The BVS explained 8% of additional variance when entered after the SVO (adjusted *R^2^* = 0.10, *p* = 0.003) but no significant amount after the PVQ-RR (adjusted *R^2^* = 0.24, *p* = 0.16). However, entering the SVO after the PVQ-RR led to a marginally significant increase in explained variance of almost 1% (adjusted *R^2^* = 0.24, *p* = 0.088). Entering the SVO on top of the BVS significantly increased the amount of explained variance by 2% (adjusted *R^2^* = 0.10, *p* = 0.008), showing that the two instruments explained largely independent variance in environmental behavior.

When entered last, the PVQ-RR explained variance on top of both other predictors (adjusted *R^2^* = 0.25, *p* < 0.001). Interestingly, the increase caused by including the SVO last was marginally significant (adjusted *R^2^* = 0.25, *p* = 0.088), but not for the BVS (adjusted *R^2^* = 0.25, *p* = 0.15). Although pro-environmental behavior is best explained by the values of the Schwartz model, respondents’ SVO explains marginally variance beyond the 19 value types.

### Hierarchical Regressions – Selective Inclusion

To probe the robustness of our findings, we conducted an additional series of hierarchical regressions. Only predictors that showed significant zero-order correlations with the outcome were included. Using this method, there was a minor change when prosocial behavior was the outcome. The BVS (suprapersonal values only) now explained significant variance above PVQ-RR (adjusted *R^2^* = 0.11, *p* = 0.023). When environmental behavior was the outcome, the SVO now significantly explained variance above and beyond the PVQ-RR (adjusted *R^2^* = 0.26, *p* = 0.027), the BVS (adjusted *R^2^* = 0.09, *p* = 0.002) and both the PVQ-RR and the BVS (adjusted *R^2^* = 0.27, *p* = 0.028). The BVS also marginally explained variance on top of the PVQ-RR (adjusted *R^2^* = 0.26, *p* = 0.052), the SVO (adjusted *R^2^* = 0.09, *p* < 0.001) and both other instruments (adjusted *R^2^* = 0.27, *p* = 0.028). While the PVQ still explains the largest amount of variance by its self (adjusted *R^2^*: PVQ-RR = 0.25; BVS = 0.07; SVO = 0.02), now all instruments explained variance above the other two measures. We found no significant changes to the results regarding prosocial behavior and mental health (see **Supplementary Materials** for detailed results).

### Multidimensional Scaling

Recall that there is controversy over the degree of overlap between the values assessed by the BVS and PVQ-RR (Gouveia et al., 2014b). To ascertain where the BVS values lie in the circumplex structure of the Schwartz model (Schwartz, 1992), we conducted MDS on responses to the value types in both value scales (i.e., 6 + 19 values). An MDS results in a graphical solution called a common space plot, which is based on the correlations between different inputs. Items with higher correlations are usually closer in space. In our case, it shows all the values and their relations based on their correlations. Values with a strong positive correlation are displayed closer together, while negatively correlated values are displayed further apart.

**FIGURE 2:**
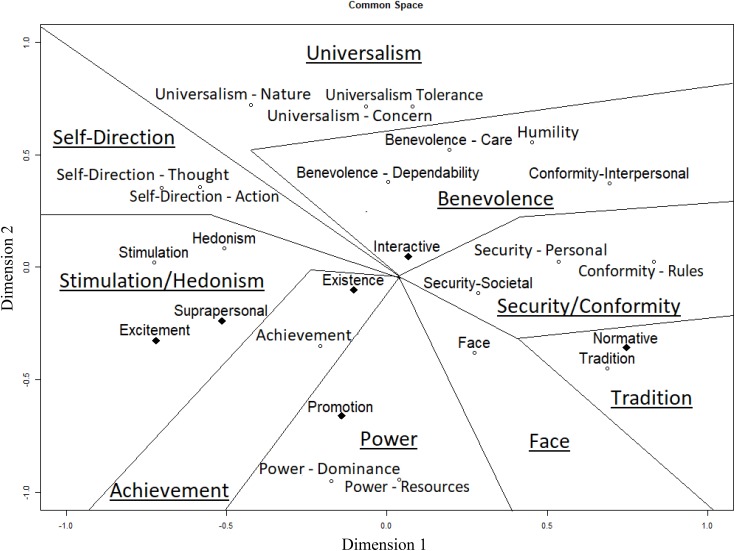
Multidimensional Scaling involving 19 PVQ-RR (marked by hollow circles) and 6 BVS values (marked by filled diamonds). Openness vs. conservation is represented as Dimension 1 (variables in the lower part of Dimension 1 indicate higher openness). Self-enhancement vs. self-transcendence is represented as Dimension 2 (variables in the lower part of Dimension 2 indicate higher self-enhancement).

Because this analysis included the BVS values with the PVQ-RR values, it is not surprising that the high Stress-1 parameter of 0.246 indicated a relatively poor fit. Nonetheless, the graphical output (**Figure 2**) generally replicated the structure of the Schwartz Model. We tested this structure by creating the borders between values described by Schwartz, while allowing the borders to kink once (Bilsky and Janik, 2010; Bilsky et al., 2011). Note that, as far as the Schwartz values are concerned, small deviations in neighboring locations (such as humility included in benevolence or hedonism included in stimulation) do not constitute a major problem. However, it is noteworthy that conformity and tradition values do not form two independent regions in the same quadrant. The conformity values can instead be found in both the security and the benevolence region.

Excitement and suprapersonal were placed in the stimulation value region, while interactive values were placed closer to benevolence values. Simultaneously, both existence, and promotion values fall into the regions of self-enhancement values (achievement and power, respectively), while normative values are related to the neighboring tradition values. Gouveia et al. (2014b) classify these values as survival values, representing the expression of lower-level needs (Maslow, 1943). The similarity in their motivational structure makes it plausible for these values to share a common region, as Schwartz (1992) suggests. Common space plots for the 19 PVQ-RR and the 6 BVS value types can be found in the **Supplementary Materials** (see footnote 1).

## Discussion

We set out to compare the ability of seven value measures to predict behavior in three important research domains: prosocial behavior, health, and the environment. However, because of low reliability issues for four of the measures, we included only three value measures (the PVQ-RR, the SVO, and the BVS) in our final analysis. Consistent with previous research using questionnaires based on the Schwartz Model (Bond and Chi, 1997; Krystallis et al., 2008; Bobowik et al., 2011), the PVQ-RR explained significant proportions of variance in prosocial behavior, mental health, and pro-environmental behavior. Respondents’ BVS values only predicted their prosocial behavior and pro-environmental behavior, while their SVO only predicted pro-environmental behavior. The hierarchical regressions showed that the PVQ-RR explained additional variance when the BVS or SVO were entered first, but these measures mostly failed to account for additional variance beyond the PVQ-RR. Overall, the PVQ-RR values not only showed the largest zero-order correlations with all three outcome variables, but they also explained the largest amount of variance in all three outcomes.

**FIGURE 3:**
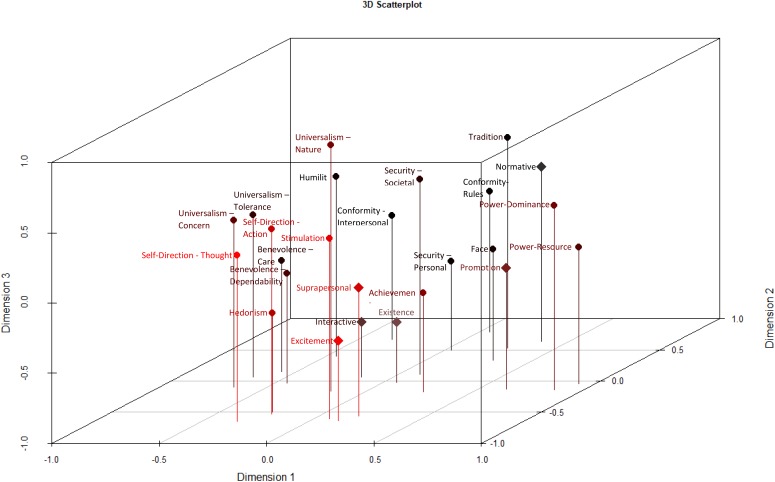
3D scatterplot depicting the results of MDS allowing for three dimensions. Dimension 1 represents Self-Enhancement (high-scores) vs. Self-Transcendence (low-scores). Dimension 2 represents Conservation (high-Scores) vs. Openness (low-scores). Dimension 3 likely represents the question formats of the different instruments, the PVQ-RR (high-scores) vs. the BVS (low-scores).

The previous discussions of conceptual overlap between the values assessed in the BVS and the PVQ-RR (Gouveia et al., 2014b; Schwartz, 2014) led us to also subject the values of the PVQ-RR and the BVS to a MDS analysis. The results replicated the Schwartz model well, with a few minor exceptions. Some BVS values clustered with PVQ-RR values possessing similar content (promotion values with power values, normative values with security values, excitement values with stimulation values), whereas other BVS values were somewhat out of place (suprapersonal values with stimulation values). Despite broadly fitting into Schwartz’s model, the BVS values retained the structure hypothesized by Gouveia et al. (2014a), with the values keeping their positions as expressions of thriving or survival needs. Furthermore, the BVS values were mostly in the hypothesized order according to the goals they represent: values representing personal goals were positioned at opposite ends from values representing social goals, with values representing central goals between them. The only exception from this rule were existence and promotion values, which appeared in reversed order. Thus, we conclude from these findings that the two value measures tap overlapping motivational space, but cluster the values in segments with different meanings. To ensure that the slight deviations from both hypothesized value structures are not due to them sharing a common space, we also subjected Schwartz’s and Gouveia’s values to two separate MDS analyses (see **Supplementary Figures S1, S2**; see footnote 1). The PVQ-RR value model did not change significantly. In Schwartz’s value model, benevolence and universalism values (except for universalism–nature) formed a single dimension. The Stress-1 value was still high at 0.23. Looking at the Gouveia’s values, changes included the values being more distanced from one another and the Stress-1 value being lower (but still unacceptably high) at 0.17. The division of the values according to their underlying need is also less clear, since suprapersonal values are clustered in with values representing survival needs, while normative values are grouped with values representing thriving needs. We further tested whether the values would fit better in a three- or four-dimensional space than a two-dimensional space. As shown in **Figure 3**, a third dimension might represent various differences in measurement between the BVS and PVQ-RR. The lowest scores on this third dimension are all occupied by BVS Values (Existence, Excitement and Interactive Values), while the highest scores belong to PVQ-RR Values (Universalism–Nature, Tradition and Security–Societal). The fit of the three-dimensional solution reduced the Stress-1 value from 0.25 to 0.16, and a four-dimensional solution reduced Stress-1 further to 0.11. However, at this point, the only distinguishable features retained in the 2-dimensional common space plot, were the four larger value domains of self-enhancement, self-transcendence, openness and conservation (**Supplementary Figures S3, S4**).

Aside from these general findings, which were the focus of our data collection, there were interesting patterns for each of the three behavioral domains. For prosocial behavior, the importance of prosocial goals (i.e., the self-transcendence values of the PVQ-RR and the suprapersonal values of the BVS) was the best predictor. Contrary to our expectations, however, a prosocial value orientation was not related to more frequent prosocial behavior. This finding contradicts the assumption that the construct measured by the SVO is actually a preference for prosocial behavior, rather than merely equal outcomes. After all, there is no option in the SVO allowing the participant to assign the other person more points than themselves. The power in our sample was sufficient to detect medium sized effects, and therefore we suggest that either the associations are small or that this result is attributable to the nature of prosocial behavior and how it was assessed here. Furthermore, prosocial behavior can have multiple goals (the same goes for pro-environmental behavior; Joireman et al., 2004). Besides prosocial goals, helpful behavior increases social status and reputation (Hardy and Van Vugt, 2006), which makes it valuable for people who pursue social dominance and achievement. An indication that this was indeed the case in our sample comes from the positive correlation between prosocial behavior and power–dominance, and the lack of a correlation with power–resources. The self-report measure of “altruistic” behavior may tap domains of prosociality wherein this motive mixing is likely (example items in the scale we used might be: “I have helped a stranger to push a car” or “I have helped an acquaintance move households,” both were highly correlated with Power–Dominance, *r* = 0.25 and *r* = 0.21, respectively. Simultaneously, these items showed a weaker association with Universalism–Nature, at most: *r* = 0.15 and *r* = 0.12).

The only significant predictor of mental health was the PVQ-RR. Coincidentally, past research shows the relationship between values and well-being to be complex (Bobowik et al., 2011, for an overview Boer, 2017). One of two prominent theories in the field, an account based on self-determination theory (Ryan and Deci, 2000), postulates that there are healthy values, such as self-transcendence and openness values, which are directly supportive of well-being. Our results find limited support for this position. Both PVQ-RR universalism and BVS suprapersonal values are at best marginally correlated with mental health. The only values significantly related to well-being were interpersonal conformity and saving face, but both are conservation values. A possible explanation comes from the second prominent theory regarding values and well-being: the person–environment congruence position (Sagiv and Schwartz, 2000). This view states that pursuing the values that are socially accepted in the person’s environment leads to increased well-being. Theoretically, conformity and saving face relates to these needs more directly. An alternative interpretation might be that people who value face are unlikely to admit to prevailing mental health issues. In this case, our findings would not represent an actual connection between valuing face and mental health, but rather showcase the tendency of individuals who value face to hide or deny undesirable aspects of themselves.

Interestingly, all three value measures significantly explained variance in pro-environmental behavior, although the PVQ-RR nature value showed the highest correlation with pro-environmental behavior. All universalism values from the PVQ-RR were reliably correlated with pro-environmental behavior; as were both self-direction values. As with prosocial behavior, the suprapersonal value dimension of the BVS significantly predicted pro-environmental behavior. Hierarchical regressions showed that the BVS did not explain variance on top of the PVQ-RR, indicating that the motivation tapped by suprapersonal values can be found in the values of the Schwartz model. The SVO explained pro-environmental behavior because respondents who made more prosocial choices were more likely to report more frequent pro-environmental behavior, whereas respondents who made more pro-self (especially competitive) choices were less likely to report pro-environmental behavior.

Our results show the PVQ-RR values are the best predictors of all three outcome measures. One explanation for this might be that the PVQ-RR items overlaps semantically more with the outcome measures than the BVS items. Specifically, recall that the PVQ-RR asks respondents to rate how similar they perceive themselves to be to a person, while all they know about that person is that a certain behavior is important to the individual (e.g., “It is important for her/him to care for nature”). Although there are some items that describe people who merely strive to be something (e.g., “It is important to her/him to be wealthy”), the more behavior-focused nature of the comparisons in the PVQ-RR might also contribute to stronger relations with behavior. Indeed, research by Arnulf et al. (2014) demonstrated how predictor–outcome similarity in meaning can influence the relationship between two measures.

A limitation of this study is that we used the cultural-level inventory VSM instead of the individual level CVSCALE to measure Hofstede’s cultural dimensions (Yoo et al., 2011). The latter scale is designed to tap the same values as the VSM, but on an individual level. This choice may at least partly explain the low reliabilities of the VSM in our sample.

As discussed above, it should also be noted that while the PVQ-RR and the BVS measure value importance, this is less clear with the SVO. The measure was described to portray “stable preferences of outcomes for oneself and others” (Van Lange et al., 1997, p. 773; for similar definition, see for example, McClintock and Liebrand, 1988). However, it is worth noting that choices in the task could also reflect social norms or habits.

In summary, our results demonstrate that the PVQ-RR is the best predictor of all three types of behavior. While the BVS and the SVO significantly explained variance in some of the outcome variables, the PVQ-RR explained variance above and beyond both in almost every case. These findings provide empirical support for the growing use of Schwartz’s scale as a predictor of value-relevant attitudes and behavior.

## Author’s Note

The Introduction partly duplicates the Introduction of the PhD-thesis of the first author (Hanel, 2016).

## Ethics Statement

The studywas approved by the ethics committee of the School of Psychology, Cardiff University. Participants provided online informed consent and were debriefed and thanked at the end.

## Author Contributions

PH: conceptualized. PH and GM: designed the study. LL and PH: data analyses and visualizations. PH, LL, and GM: wrote the original draft and the revised draft.

## Conflict of Interest Statement

The authors declare that the research was conducted in the absence of any commercial or financial relationships that could be construed as a potential conflict of interest.

## Footnotes

1 https://osf.io/jd6ze/
