# Supplementary material for: An Empirical Comparison of Human Value Models
Source: Front Psychol. 2018 Sep 25;9:1643. doi: 10.3389/fpsyg.2018.01643 (PMC6167453; doi:10.3389/fpsyg.2018.01643)
Supplement: Supplementary file 1 [file Data_Sheet_1.docx]

**An empirical comparison of human value models**

**Online Supplemental Materials**

## Supplemental Figures


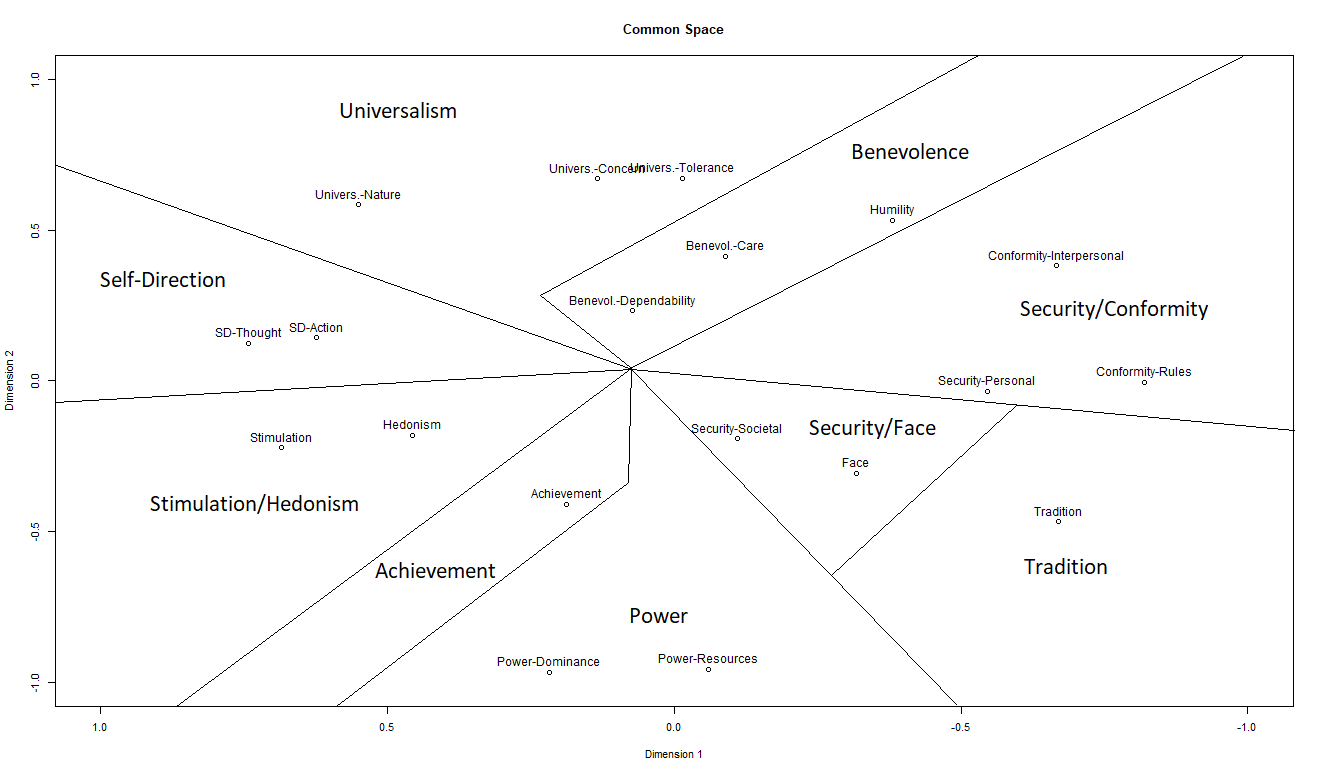


*Figure S1*. Common space plot of Schwartz’s revised 19-value type model.


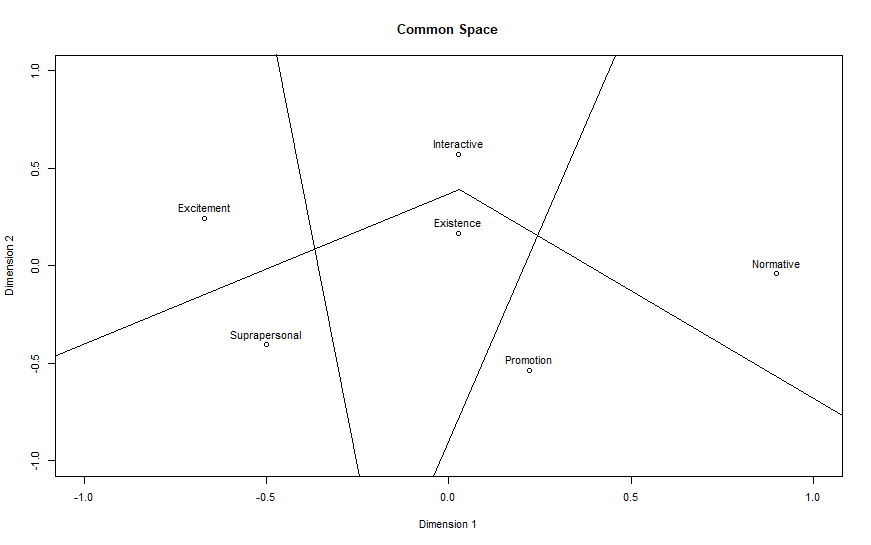


*Figure S2*. Common space plot of Gouveia’s functional theory model.


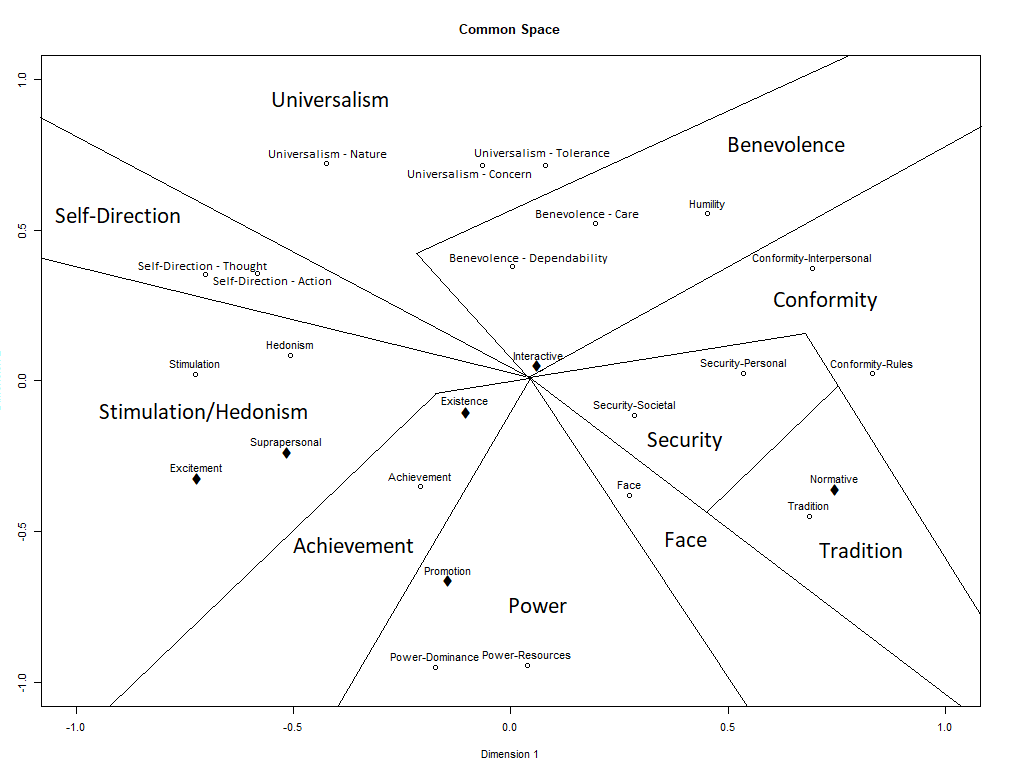


*Figure S3*. The two meaningful dimensions of the 3-dimensional MDS common space plot.


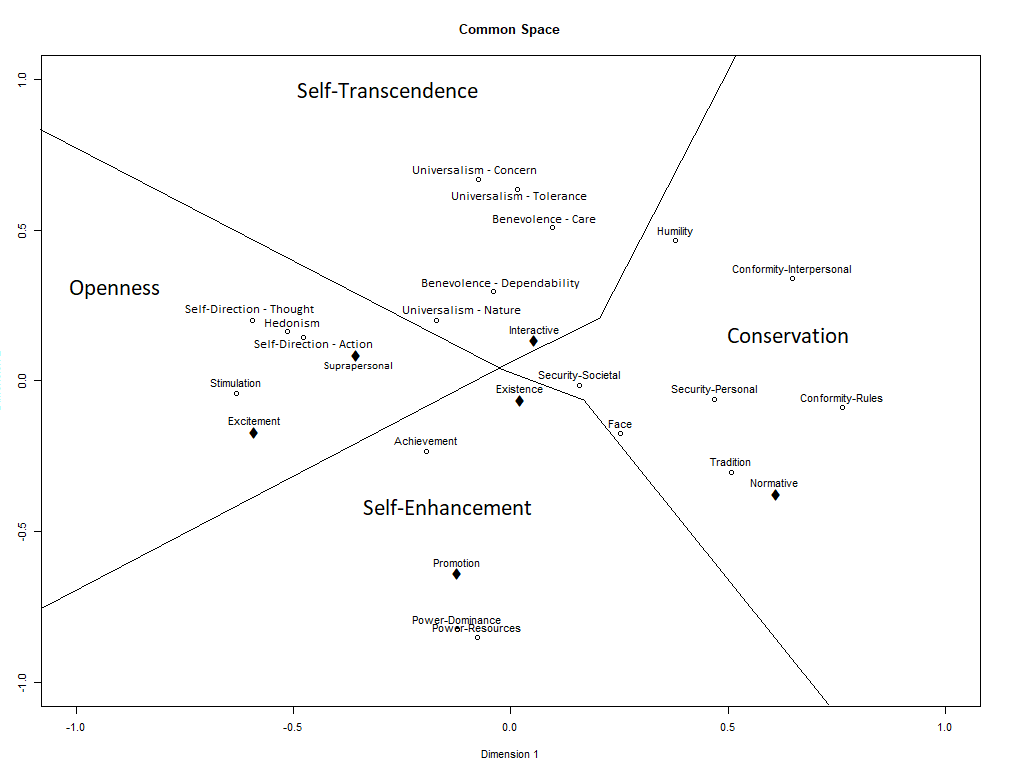


*Figure S4*. The two meaningful dimensions of the 4-dimensional MDS common space plot.

## Hierarchical Regressions – Selective Inclusion

| **Table 1.** Altruism predicted by PVQ + SVO + BVS. Hierarchical Regression | | | | | | | |
| --- | --- | --- | --- | --- | --- | --- | --- |
|  | Adj. R^2^ | Res.Df | RSS | Df | SSQ | F | p |
| PVQ + | .12 | 224 | 54.118 |  |  |  |  |
| SVO | .12 | 223 | 54.079 | 1 | .04 | .16 | .69 |
| SVO + BVS | .14 | 222 | 52.858 | 1 | 1.22 | 5.13 | .026* |
| *Note*. PVQ = Self Direction – Action, Stimulation, Power – Dominance, Security – Societal, Tradition, Humility, Universalism – Nature, Universalism – Concern, Universalism – Tolerance, Benevolence - Care, Benevolence - Dependability; SVO = SVO; BVS = Suprapersonal Significance level: ^t^p < .1, *p < .05, **p < .01, ***p < .001 | | | | | | | |

| **Table 2.** Altruism predicted by PVQ + BVS + SVO. Hierarchical Regression | | | | | | | |
| --- | --- | --- | --- | --- | --- | --- | --- |
|  | Adj. R^2^ | Res.Df | RSS | Df | SSQ | F | p |
| PVQ + | .12 | 224 | 54.118 |  |  |  |  |
| BVS | .14 | 223 | 52.863 | 1 | 1.25 | 5.27 | .023* |
| BVS + SVO | .14 | 222 | 52.858 | 1 | .01 | .02 | .883 |
| *Note*. PVQ = Self Direction – Action, Stimulation, Power – Dominance, Security – Societal, Tradition, Humility, Universalism – Nature, Universalism – Concern, Universalism – Tolerance, Benevolence - Care, Benevolence - Dependability; SVO = SVO; BVS = Suprapersonal Significance level: ^t^p < .1, *p < .05, **p < .01, ***p < .001 | | | | | | | |

| **Table 3.** Altruism predicted by SVO + PVQ + BVS. Hierarchical Regression | | | | | | | |
| --- | --- | --- | --- | --- | --- | --- | --- |
|  | Adj. R^2^ | Res.Df | RSS | Df | SSQ | F | p |
| SVO + | <.01 | 234 | 64.804 |  |  |  |  |
| PVQ | .12 | 223 | 54.079 | 11 | 10.73 | 4.1 | < .001*** |
| PVQ + BVS | .14 | 222 | 52.858 | 1 | 1.22 | 5.13 | .025* |
| *Note*. PVQ = Self Direction – Action, Stimulation, Power – Dominance, Security – Societal, Tradition, Humility, Universalism – Nature, Universalism – Concern, Universalism – Tolerance, Benevolence - Care, Benevolence - Dependability; SVO = SVO; BVS = Suprapersonal Significance level: ^t^p < .1, *p < .05, **p < .01, ***p < .001 | | | | | | | |

| **Table 4.** Altruism predicted by SVO + BVS + PVQ. Hierarchical Regression | | | | | | | |
| --- | --- | --- | --- | --- | --- | --- | --- |
|  | Adj. R^2^ | Res.Df | RSS | Df | SSQ | F | p |
| SVO + | <.01 | 234 | 64.804 |  |  |  |  |
| BVS | .06 | 233 | 60.422 | 1 | 4.38 | 18.40 | < .001*** |
| BVS + PVQ | .14 | 222 | 52.858 | 11 | 7.56 | 2.89 | .001** |
| *Note*. PVQ = Self Direction – Action, Stimulation, Power – Dominance, Security – Societal, Tradition, Humility, Universalism – Nature, Universalism – Concern, Universalism – Tolerance, Benevolence - Care, Benevolence - Dependability; SVO = SVO; BVS = Suprapersonal Significance level: ^t^p < .1, *p < .05, **p < .01, ***p < .001 | | | | | | | |

| **Table 5.** Altruism predicted by BVS + PVQ + SVO. Hierarchical Regression | | | | | | | |
| --- | --- | --- | --- | --- | --- | --- | --- |
|  | Adj. R^2^ | Res.Df | RSS | Df | SSQ | F | p |
| BVS + | .06 | 234 | 60.479 |  |  |  |  |
| PVQ | .14 | 223 | 52.863 | 11 | 7.62 | 2.91 | .001** |
| PVQ + SVO | .14 | 222 | 52.858 | 1 | .01 | .02 | .883 |
| *Note*. PVQ = Self Direction – Action, Stimulation, Power – Dominance, Security – Societal, Tradition, Humility, Universalism – Nature, Universalism – Concern, Universalism – Tolerance, Benevolence - Care, Benevolence - Dependability; SVO = SVO; BVS = Suprapersonal Significance level: ^t^p < .1, *p < .05, **p < .01, ***p < .001 | | | | | | | |

| **Table 6.** Altruism predicted by BVS + SVO + PVQ. Hierarchical Regression | | | | | | | |
| --- | --- | --- | --- | --- | --- | --- | --- |
|  | Adj. R^2^ | Res.Df | RSS | Df | SSQ | F | p |
| BVS + | .06 | 234 | 60.479 |  |  |  |  |
| SVO | .06 | 233 | 60.422 | 1 | .06 | .24 | .627 |
| SVO + PVQ | .14 | 222 | 52.858 | 11 | 7.56 | 2.89 | .001** |
| *Note*. PVQ = Self Direction – Action, Stimulation, Power – Dominance, Security – Societal, Tradition, Humility, Universalism – Nature, Universalism – Concern, Universalism – Tolerance, Benevolence - Care, Benevolence - Dependability; SVO = SVO; BVS = Suprapersonal Significance level: ^t^p < .1, *p < .05, **p < .01, ***p < .001 | | | | | | | |

| **Table 7.** General Health predicted by PVQ + SVO + BVS. Hierarchical Regression | | | | | | | |
| --- | --- | --- | --- | --- | --- | --- | --- |
|  | Adj. R^2^ | Res.Df | RSS | Df | SSQ | F | p |
| PVQ + | .1 | 232 | 50.805 |  |  |  |  |
| SVO | .1 | 231 | 50.718 | 1 | .09 | .4 | .528 |
| SOV + BVS | .11 | 230 | 50.066 | 1 | .65 | 2.99 | .085^t^ |
| *Note*. PVQ = Hedonism, Face, Conformity – Interpersonal; SVO = SVO; BVS = Suprapersonal  Significance level: ^t^p < .1, *p < .05, **p < .01, ***p < .001 | | | | | | | |

| **Table 8.** General Health predicted by PVQ + BVS + SVO. Hierarchical Regression | | | | | | | |
| --- | --- | --- | --- | --- | --- | --- | --- |
|  | Adj. R^2^ | Res.Df | RSS | Df | SSQ | F | P |
| PVQ + | .1 | 232 | 50.805 |  |  |  |  |
| BVS | .11 | 231 | 50.148 | 1 | .66 | 3.02 | .084^t^ |
| BVS + SVO | .11 | 230 | 50.066 | 1 | .08 | .37 | .542 |
| *Note*. PVQ = Hedonism, Face, Conformity – Interpersonal; SVO = SVO; BVS = Suprapersonal  Significance level: ^t^p < .1, *p < .05, **p < .01, ***p < .001 | | | | | | | |

| **Table 9.** General Health predicted by SVO + PVQ + BVS. Hierarchical Regression | | | | | | | |
| --- | --- | --- | --- | --- | --- | --- | --- |
|  | Adj. R^2^ | Res.Df | RSS | Df | SSQ | F | p |
| SVO + | <.01 | 234 | 57.163 |  |  |  |  |
| PVQ | .09 | 231 | 50.718 | 3 | 6.45 | 9.87 | < .001*** |
| PVQ + BVS | .11 | 230 | 50.066 | 1 | .65 | 2.99 | .085^t^ |
| *Note*. PVQ = Hedonism, Face, Conformity – Interpersonal; SVO = SVO; BVS = Suprapersonal  Significance level: ^t^p < .1, *p < .05, **p < .01, ***p < .001 | | | | | | | |

| **Table 10.** General Health predicted by SVO + BVS + PVQ. Hierarchical Regression | | | | | | | |
| --- | --- | --- | --- | --- | --- | --- | --- |
|  | Adj. R^2^ | Res.Df | RSS | Df | SSQ | F | p |
| SVO + | <.01 | 234 | 57.163 |  |  |  |  |
| BVS | .01 | 233 | 56.16 | 1 | 1 | 4.61 | .033* |
| BVS + PVQ | .11 | 230 | 50.066 | 3 | 6.09 | 9.33 | < .001*** |
| *Note*. PVQ = Hedonism, Face, Conformity – Interpersonal; SVO = SVO; BVS = Suprapersonal  Significance level: ^t^p < .1, *p < .05, **p < .01, ***p < .001 | | | | | | | |

| **Table 11.** General Health predicted by BVS + PVQ + SVO. Hierarchical Regression | | | | | | | | | | | | |  |
| --- | --- | --- | --- | --- | --- | --- | --- | --- | --- | --- | --- | --- | --- |
|  | Adj. R^2^ | Res.Df | | RSS | | Df | | SSQ | | F | | p |  |
| BVS + | .01 | 234 | | 56.168 | |  | |  | |  | |  |  |
| PVQ | .11 | 231 | | 50.148 | | 3 | | 6.02 | | 9.22 | | < .001*** |  |
| PVQ + SVO | .11 | 230 | | 50.066 | | 1 | | .08 | | .37 | | .855 |  |
| *Note*. PVQ = Hedonism, Face, Conformity – Interpersonal; SVO = SVO; BVS = Suprapersonal  Significance level: ^t^p < .1, *p < .05, **p < .01, ***p < .001 | | | | | | | | | | | | |  |
|  | | **Table 12.** General Health predicted by BVS + SVO + PVQ. Hierarchical Regression | | | | | | | | | | | |
|  | | Adj. R^2^ | | Res.Df | | RSS | | Df | | SSQ | | F | p |
| BVS + | | .01 | | 234 | | 56.168 | |  | |  | |  |  |
| SVO | | .01 | | 233 | | 56.16 | | 1 | | .01 | | .03 | .848 |
| SVO + PVQ | | .11 | | 230 | | 50.066 | | 3 | | 6.09 | | 9.33 | < .001*** |
| *Note*. PVQ = Hedonism, Face, Conformity – Interpersonal; SVO = SVO; BVS = Suprapersonal  Significance level: ^t^p < .1, *p < .05, **p < .01, ***p < .001 | | | | | | | | | | | | | |

| **Table 13.** Environmentalism predicted by PVQ + SVO + BVS. Hierarchical Regression | | | | | | | |
| --- | --- | --- | --- | --- | --- | --- | --- |
|  | Adj. R^2^ | Res.Df | RSS | Df | SSQ | F | p |
| PVQ + | .25 | 228 | 66.993 |  |  |  |  |
| SVO | .26 | 227 | 65.579 | 1 | 1.41 | 4.98 | .027* |
| SVO + BVS | .27 | 226 | 64.19 | 1 | 1.39 | 4.89 | .028* |
| *Note*. PVQ = Self-Direction – Thought, Self-Direction – Action, Conformity – Interpersonal, Humility, Universalism – Nature, Universalism – Concern, Universalism - Tolerance; SVO = SVO; BVS = Suprapersonal Significance level: ^t^p < .1, *p < .05, **p < .01, ***p < .001 | | | | | | | |

| **Table 14.** Environmentalism predicted by PVQ + BVS + SVO. Hierarchical Regression | | | | | | | |
| --- | --- | --- | --- | --- | --- | --- | --- |
|  | Adj. R^2^ | Res.Df | RSS | Df | SSQ | F | p |
| PVQ + | .25 | 228 | 66.993 |  |  |  |  |
| BVS | .26 | 227 | 65.906 | 1 | 1.09 | 3.83 | .052^t^ |
| BVS + SVO | .27 | 226 | 64.19 | 1 | 1.72 | 6.04 | .014* |
| *Note*. PVQ = Self-Direction – Thought, Self-Direction – Action, Conformity – Interpersonal, Humility, Universalism – Nature, Universalism – Concern, Universalism - Tolerance; SVO = SVO; BVS = Suprapersonal Significance level: ^t^p < .1, *p < .05, **p < .01, ***p < .001 | | | | | | | |

| **Table 15.** Environmentalism predicted by SVO + PVQ + BVS. Hierarchical Regression | | | | | | | |
| --- | --- | --- | --- | --- | --- | --- | --- |
|  | Adj. R^2^ | Res.Df | RSS | Df | SSQ | F | p |
| SVO + | .02 | 234 | 89.141 |  |  |  |  |
| PVQ | .26 | 227 | 65.579 | 7 | 23.56 | 11.85 | < .001*** |
| PVQ + BVS | .27 | 226 | 64.19 | 1 | 1.39 | 4.89 | .028* |
| *Note*. PVQ = Self-Direction – Thought, Self-Direction – Action, Conformity – Interpersonal, Humility, Universalism – Nature, Universalism – Concern, Universalism - Tolerance; SVO = SVO; BVS = Suprapersonal Significance level: ^t^p < .1, *p < .05, **p < .01, ***p < .001 | | | | | | | |

| **Table 16.** Environmentalism predicted by SVO + BVS + PVQ. Hierarchical Regression | | | | | | | |
| --- | --- | --- | --- | --- | --- | --- | --- |
|  | Adj. R^2^ | Res.Df | RSS | Df | SSQ | F | p |
| SVO + | .02 | 234 | 89.141 |  |  |  |  |
| BVS | .09 | 233 | 82.17 | 1 | 6.97 | 24.54 | < .001*** |
| BVS + PVQ | .27 | 226 | 64.19 | 7 | 17.98 | 9.04 | < .001*** |
| *Note*. PVQ = Self-Direction – Thought, Self-Direction – Action, Conformity – Interpersonal, Humility, Universalism – Nature, Universalism – Concern, Universalism - Tolerance; SVO = SVO; BVS = Suprapersonal Significance level: ^t^p < .1, *p < .05, **p < .01, ***p < .001 | | | | | | | |

| **Table 17.** Environmentalism predicted by BVS + PVQ + SVO. Hierarchical Regression | | | | | | | |
| --- | --- | --- | --- | --- | --- | --- | --- |
|  | Adj. R^2^ | Res.Df | RSS | Df | SSQ | F | p |
| BVS + | .07 | 234 | 84.962 |  |  |  |  |
| PVQ | .26 | 227 | 65.906 | 7 | 19.06 | 9.58 | < .001*** |
| PVQ + SVO | .27 | 226 | 64.19 | 1 | 1.72 | 6.04 | .015* |
| *Note*. PVQ = Self-Direction – Thought, Self-Direction – Action, Conformity – Interpersonal, Humility, Universalism – Nature, Universalism – Concern, Universalism - Tolerance; SVO = SVO; BVS = Suprapersonal Significance level: ^t^p < .1, *p < .05, **p < .01, ***p < .001 | | | | | | | |

| **Table 18.** Environmentalism predicted by BVS + SVO + PVQ. Hierarchical Regression | | | | | | | |
| --- | --- | --- | --- | --- | --- | --- | --- |
|  | Adj. R^2^ | Res.Df | RSS | Df | SSQ | F | p |
| BVS + | .07 | 234 | 84.962 |  |  |  |  |
| SVO | .09 | 233 | 82.17 | 1 | 2.79 | 9.83 | .002** |
| SVO + PVQ | .27 | 226 | 64.19 | 7 | 17.98 | 9.04 | < .001*** |
| *Note*. PVQ = Self-Direction – Thought, Self-Direction – Action, Conformity – Interpersonal, Humility, Universalism – Nature, Universalism – Concern, Universalism - Tolerance; SVO = SVO; BVS = Suprapersonal Significance level: ^t^p < .1, *p < .05, **p < .01, ***p < .001 | | | | | | | |
